# Death by Necrosis: The Early Stages of Type 1 Diabetes

**DOI:** 10.1371/journal.pmed.0030051

**Published:** 2005-12-20

Diabetes mellitus is a major global health problem. The most common form of diabetes—90%–95% of cases—is type 2 or non-insulin-dependent diabetes, which affects mainly adults. The remaining cases are type 1 or insulin-dependent diabetes. Also known as juvenile diabetes, type 1 diabetes usually develops before the age of 30. It is an autoimmune disease in which the insulin-producing or β-cells of the pancreatic islets of Langerhans (groups of specialized cells that regulate blood sugar levels) are destroyed by lymphocytes. Type 1 diabetes affects about one in 500 American children and adolescents; the only treatment is daily insulin injections.

β-cell death is the hallmark of type 1 diabetes. An early phase of β-cell death, probably triggered by environmental factors (for example, viral infection) in genetically susceptible individuals, releases β-cell-specific antigens; subsequently, T lymphocytes that specifically recognize these antigens mediate widespread β-cell killing. John Corbett and colleagues believe that by studying the early phase of β-cell death, it may be possible to find ways to prevent this destructive autoimmunity from developing in individuals with a family history of type 1 diabetes.

Cytokines, chemical messengers produced by lymphocytes and macrophages, are thought to contribute to the loss of β-cell function and viability early in autoimmune diabetes. The effect of cytokines on β-cell function is mediated by nitric oxide (NO), but it is not clear if the same is true for β-cell death. In their study, Corbett and colleagues asked whether NO mediates the death of rat β-cells induced in vitro by the macrophage-derived cytokine interleukin-1 (IL-1), and whether the cells are killed by apoptosis or necrosis, two different mechanisms of cell death. Apoptosis, or programmed cell death, is a highly organized process that minimizes the leakage of cell contents and the development of inflammation. Necrosis is much less tidy: the dying cells swell and burst, releasing their contents into the extracellular space where they cause inflammation.

The researchers report that 24–48 hours treatment with IL-1 reduced the viability of rat β-cells from two sources—an insulinoma cell line and islets. Then, by inhibiting NO synthesis or by adding an NO donor, they provide evidence that IL-1-induced death of β-cells is mediated in part by NO production. Turning to the mechanism of β-cell death, the researchers show that IL-1 treatment failed to activate caspase 3—an enzyme required for apoptosis—in β-cells, and that a caspase-3 inhibitor did not attenuate IL-1 induced β-cell death. Another marker of apoptotic cell death—lipid accumulation on the cell surface—was also missing in β-cells treated with IL-1. [Fig pmed-0030051-g001]

**Figure pmed-0030051-g001:**
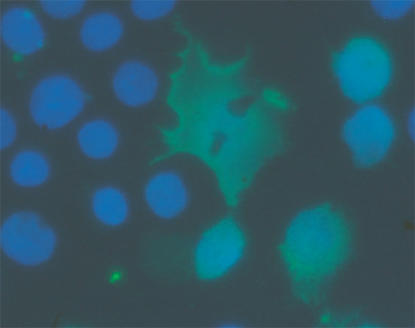
IL-1 causes nuclear membrane breakdown in rat insulinoma cells

Having discounted death by apoptosis, the researchers then show that IL-1 stimulated the release of HMGB1 (a chromatin-binding protein that is released by cells undergoing necrosis but not apoptosis) by rat β-cells. Finally, because human β-cells behave somewhat differently from rat β-cells, the researchers demonstrate that a combination of cytokines (including IL-1) stimulated HMGB1 release from some preparations of human islets in an NO-dependent manner.

Overall, the authors conclude that macrophage-derived cytokines may participate in the early stages of type 1 diabetes by inducing necrotic death in β-cells. Other researchers believe that apoptotic cell death is more important in these early stages, particularly in human cells. But, based on their results, Corbett and colleagues speculate that cytokine induction of necrosis could kick start type 1 diabetes by causing the release of both β-cell antigens and HMGB1, which stimulates inflammatory responses. Although these findings need to be confirmed within the context of the human pancreas—what cells do in vitro may not reflect what happens in the body—they provide new insights into the early stages of type 1 diabetes that could suggest ways to prevent or stop its development.

